# Multiple Myeloma in an Agricultural Worker Exposed to Pesticides

**DOI:** 10.7759/cureus.4762

**Published:** 2019-05-28

**Authors:** Elizabeth Packard, Zainab Shahid, Andrew Groff, Ravi Patel, Rohit Jain

**Affiliations:** 1 Internal Medicine, Penn State Health Milton S. Hershey Medical Center, Hershey, USA; 2 Internal Medicine, Lake Erie College of Osteopathic Medicine, Erie, USA

**Keywords:** multiple myeloma, captan, pesticide, phthalimide, fungicide, agriculture, carcinogenic agent

## Abstract

Multiple myeloma (MM) is a neoplastic disorder characterized by the monoclonal proliferation of plasma cells in the bone marrow. It is estimated to account for only 1% of neoplastic diseases, and there is still a great deal of uncertainty about its precise etiology. Common risk factors with a proven association with MM include ionizing radiation exposure, age greater than 65, male gender, and the presence of monoclonal gammopathy of unknown significance (MGUS).More recently, research has shown that occupational exposures to pesticides also have a significant association with the development of MM. We present the case of an adult male who presented with rib pain, back pain, fevers, and progressive shortness of breath and was ultimately found to have multiple myeloma thought to be associated with occupational exposure to the pesticide captan.

## Introduction

Multiple myeloma (MM) is a malignant plasma cell disorder characterized by an aberrant expansion of plasma cells in the bone marrow that produces a monoclonal immunoglobulin [1]. One of the most common presenting symptoms is bone pain due to the proliferation of plasma cells within the bone matrix. Neoplastic plasma cells activate osteoclasts and lead to the weakening of the matrix and subsequent clinical presentation with osteopenia, osteolytic lesions, or pathologic fractures. Increased bone turnover leads to an efflux of calcium into the extracellular fluid, causing symptomatic hypercalcemia. Patients may also present with recurrent infections due to the decreased quantity of polyclonal immunoglobulins, pancytopenia (most commonly anemia) secondary to the replacement of normal hematopoietic tissue by tumor cells, and renal failure due to the accumulation of excess light chains with tubular cast formation and obstructive nephropathy [2].

The diagnosis of MM requires greater than 10% plasma cells on bone marrow biopsy and either increased monoclonal protein in the serum or urine or signs of end-organ damage [3]. Common signs of end-organ damage include bone lesions, hypercalcemia with a calcium level greater than or equal to 11.5 g/dL, renal insufficiency with a creatinine level greater than or equal to 2 mg/dL, and anemia with a hemoglobin level less than or equal to 10 g/dL [2]. Treatment with systemic chemotherapy, commonly bortezomib-based regimens, is initiated immediately upon symptom presentation or end-organ damage. Despite further research regarding effective treatments and novel advances, the prognosis remains unfavorable with a five-year survival rate of 38% [4].

While it has been established that MM is a process associated with aging, the ultimate cause of MM has yet to be elucidated. Monoclonal gammopathy of unknown significance (MGUS), an asymptomatic plasma cell dyscrasia with less than 10% plasma cells in the bone marrow, has been identified as a key component of the pathophysiology and precedes almost all cases of MM [1,4-6]. However, there is still uncertainty with regards to the exact cause of MGUS and the triggers that stimulate the progression of MGUS to MM. Research has shown that this plasma cell disorder has a genetic component, with a wide variety of chromosomal abnormalities detected in up to 90% of MM patients [2]. However, several recent American and Canadian studies have shown that environmental exposures also play a key role in the pathogenesis of MM. Of note, agricultural occupations with pesticide exposure have consistently shown to be associated with carcinogenesis and an increased risk of developing MM [2,7].

## Case presentation

A 64-year-old male with a past medical history of nephrolithiasis and hypertension presented to the emergency department with left-sided rib pain, back pain, progressive shortness of breath, and fevers over the past month. The patient was visiting from the Dominican Republic, where he worked in agriculture and used the fungicide captan. He reported an unintentional 12-pound weight loss and worsening back pain over the past month. He denied recent trauma, nausea, vomiting, chest pain, dysuria, or hematuria. He also denied ever smoking tobacco and drinking alcohol.

**Figure 1:**
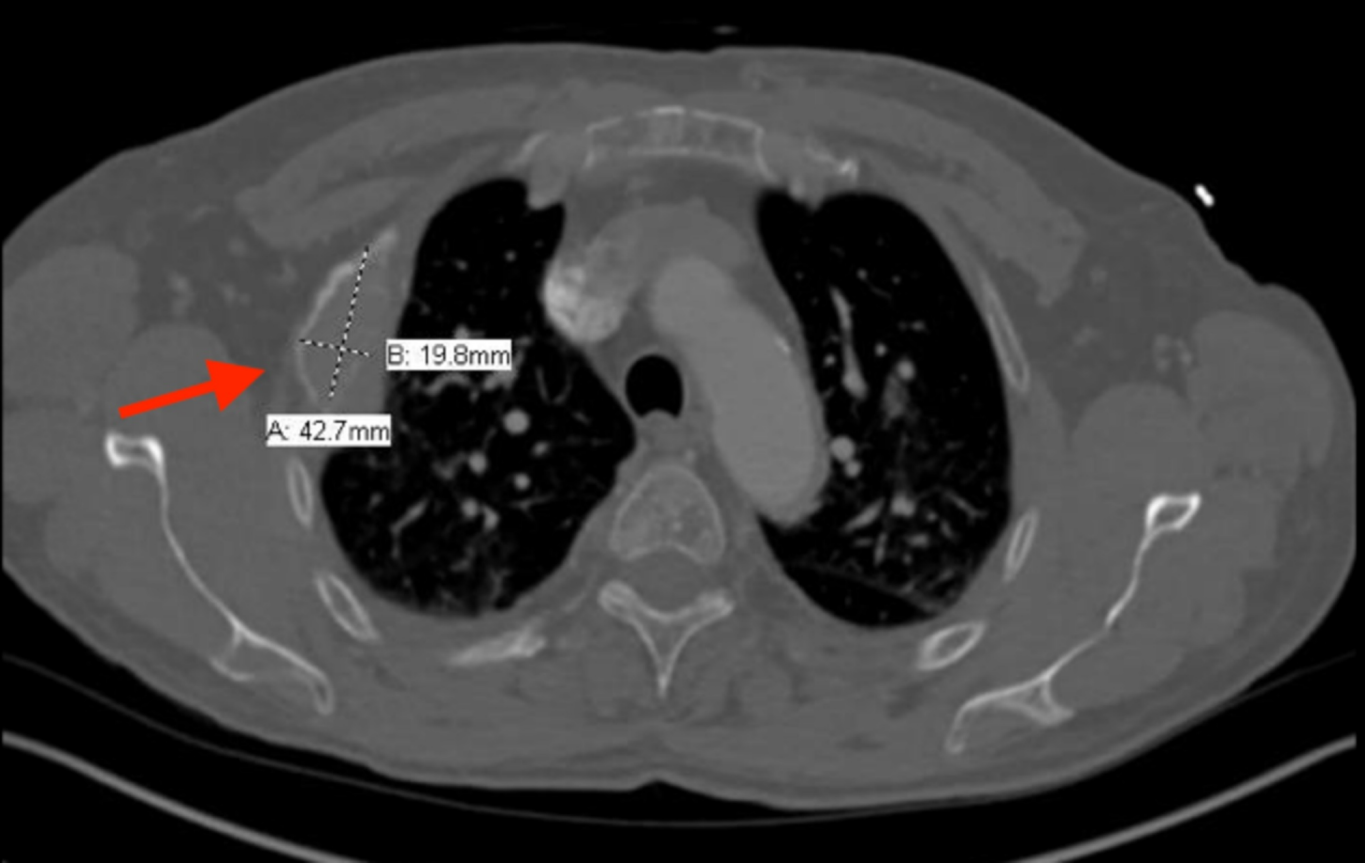
Non-contrast CT scan of the chest revealing an expansile lytic lesion involving the right rib with soft tissue involvement (red arrow) concerning for malignancy CT: Computed tomography

Upon presentation, he was in no apparent distress and vital signs revealed a temperature of 36.7°C, a pulse of 121 beats per minute, blood pressure of 148/95 mmHg, respiratory rate of 21, and oxygen saturation of 96% on room air. Physical exam was significant for left upper quadrant abdominal pain. A computed tomography (CT) scan of the abdomen revealed an expansile lytic lesion involving the right rib (Figure 1).

Given the patient’s history of unintentional weight loss and numerous lytic lesions, laboratory workup for MM was initiated. This revealed an elevated total serum protein level at 11.3 g/dL (normal range 6.4-8.3 g/dL), calcium level of 8.4 g/dL (normal range 8.4-10.2 g/dL), decreased albumin level of 2.5 mg/dL (normal range 3.5-5.2 mg/dL), and elevated b2-microglobulin level at 4.26 mg/L (0.8-2.6 mg/L). Serum-free monoclonal light chain analysis revealed elevated free kappa chains at 14.39 mg/dL (normal range 0.33-1.94 mg/dL), decreased free lambda chains at 0.56 mg/dL (normal range 0.57-2.63 mg/dL), and an elevated kappa/lambda ratio at 25.70 (normal range 0.26-1.65). Serum protein electrophoresis (SPEP) revealed an elevated monoclonal gamma immunoglobulin spike at 6,500 mg/dL (normal 0). Serum immunoglobulins revealed an elevated immunoglobulin G (IgG) level at 8,060 mg/dL (normal range 700-1,600 mg/dL) and a decreased immunoglobulin A (IgA) level at 7 mg/dL (normal range 70-400 mg/dL).

**Figure 2:**
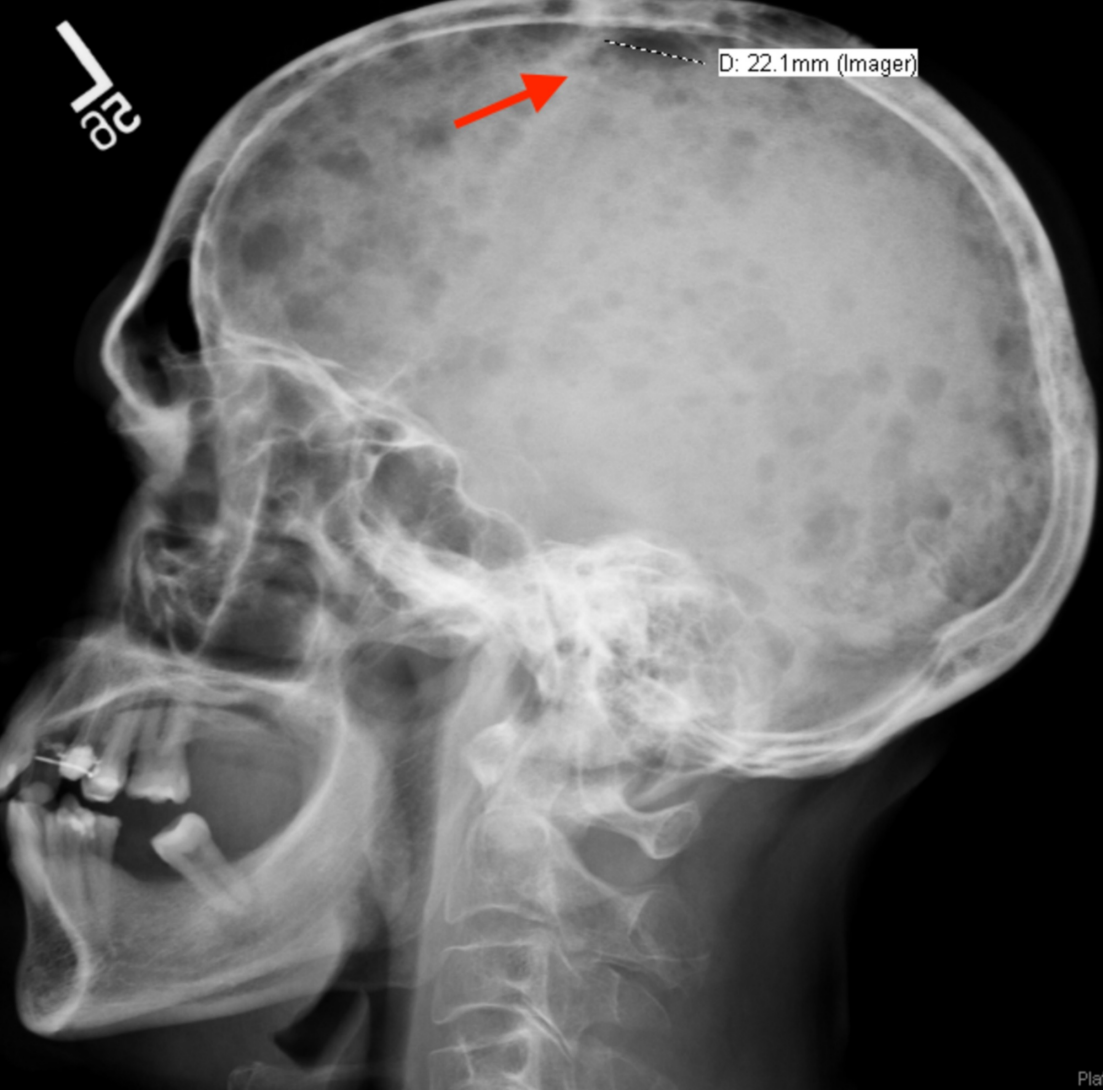
X-ray of the skull revealing a round, lytic lesion (red arrow), suggestive of multiple myeloma

**Figure 3:**
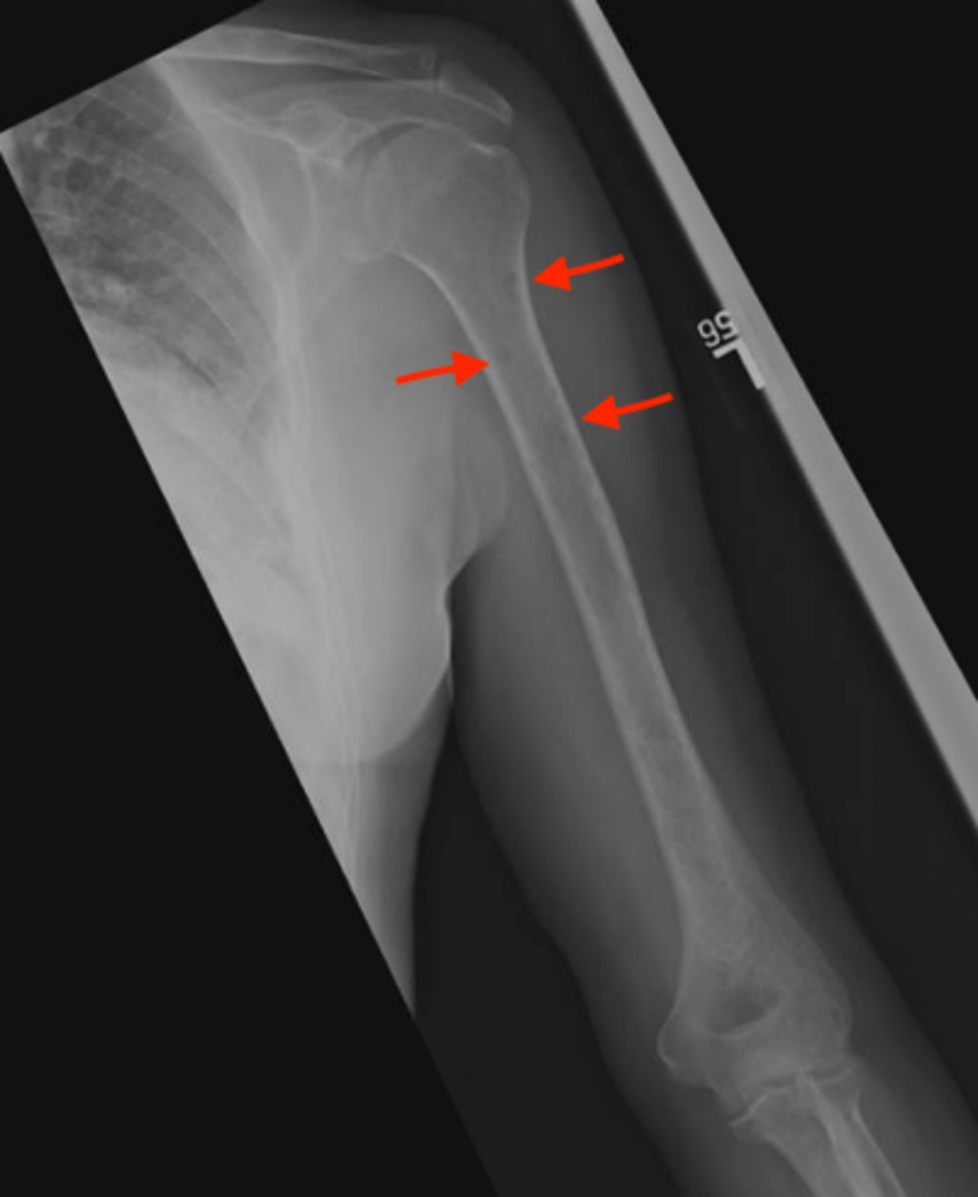
X-ray of the left humerus revealing multiple round, lytic lesions (red arrows), suggestive of multiple myeloma

A bone survey revealed multiple, round, lytic, “punched-out” lesions in the skull (Figure 2) and left humerus (Figure 3). The diagnosis of MM was confirmed with a bone marrow aspirate revealing 60% plasma cells (normal range 0%-1.2%). The patient was admitted to the hematology and oncology service for chemotherapy treatment with bortezomib and cyclophosphamide. He was discharged after one week of inpatient treatment and scheduled for outpatient chemotherapy.

## Discussion

Pesticides, including insecticides, fungicides, and herbicides, are used extensively worldwide by farmers and agricultural workers for weed destruction and pest control. Despite their widespread use, there are many documented health toxicities associated with these chemicals, ranging from a self-limited rash to cancer. Two of the most common cancers associated with pesticide use include non-Hodgkin lymphoma and leukemia [6,8]. While the etiology and pathogenesis of MM are still under investigation, recent studies have shown an elevated risk of MM with pesticide use [2,6,9-10]. For example, one case-control study collected extensive information, including occupational history from 573 persons with newly diagnosed multiple myeloma, and developed a job-exposure matrix for pesticide exposure. They found that individuals who used herbicides and fungicides on a regular basis are one and a half times more likely to develop multiple myeloma than the general population and are eight times more likely to have the associated mortality [6]. Another study found that among a subset of agricultural workers who used pesticides on a regular basis, the prevalence of MGUS was approximately 7%, which is double the prevalence when compared to the general population [9]. There are several hypotheses regarding pesticide-induced carcinogenesis, including increased production of reactive oxygen species contributing to DNA damage, immunotoxicity to helper T-cells, and direct genotoxicity and the subsequent chromosomal aberrations [11].

Of the various classes of pesticides, insecticides, including carbamates and organochlorines (e.g. dichlorodiphenyltrichloroethane, cyclodienes), have classically shown the strongest association with the development of MM [5,9]. Patients with insecticide exposure have been shown to have a two- to five-fold increased risk of developing MGUS and/or MM [5,11-12]. However, individual chemicals within the various classes have also been shown to have carcinogenic properties. One of these particular chemicals is the fungicide captan, to which our patient was exposed. Captan is a phthalimide agricultural fungicide that has been used commercially for tree fruits for over 60 years. There has been a great deal of debate worldwide regarding its toxicity. In the United States, the EPA or environmental protection agency classifies captan as “not likely carcinogenic to humans,” whereas the European commission classifies it as “suspected of causing cancer” [10]. Due to this controversy and given its extensive use, several recent studies have investigated the potential toxicity of captan and demonstrated a consistent increased risk of MM with captan exposure [5,10-12]. An analysis of three case-control studies comparing 547 patients with multiple myeloma and 2,700 controls found that less than 17.5 lifetime days of exposure to captan was associated with a more than three-fold increase in the risk of developing multiple myeloma [11]. Proposed mechanisms support the involvement of at least two separate pathways, including direct genotoxic effects with DNA strand breaks in mammalian cells exposed to captan, and increased activation of caspases leading to increased cell production within the bone marrow and subsequent neoplastic transformation [12].

While some pesticides with demonstrated carcinogenic properties have been restricted or banned, there are many that are still available for sale and use, particularly in developing countries. For example, dichlorodiphenyltrichloroethane (DDT) was banned in the United States in 1972, but it was used in China until 2007. It is still used today in India and in countries in Africa and South America to reduce the risk of malaria [11]. The fungicide captan is also still used widely for agricultural purposes across the world, despite the European commission classifying it as “suspected of causing cancer.” Although captan is amongst the lesser known carcinogenic chemicals, it is important to consider MM in patients presenting with consistent signs and symptoms and associated history of captan exposure.

## Conclusions

We present a rare case of multiple myeloma thought to be associated with occupational exposure to the fungal pesticide, captan. While the underlying etiologies of multiple myeloma are still under investigation, there appear to be both genetic and environmental exposure components that lead to an increased risk. Given the poor prognosis of multiple myeloma even with treatment, we place emphasis on the importance of a high index of suspicion for multiple myeloma in patients with constitutional symptoms and exposure to pesticides.
